# Towards New Broader Spectrum Pneumococcal Vaccines: The Future of Pneumococcal Disease Prevention

**DOI:** 10.3390/vaccines2010112

**Published:** 2014-02-14

**Authors:** Lucia H. Lee, Xin-Xing Gu, Moon H. Nahm

**Affiliations:** 1 Center for Biologics Evaluation and Research, Food and Drug Administration, Rockville, MD 20852, USA; 2 National Institute of Allergy and Infectious Diseases, Bethesda, MD 20892, USA; E-Mail: guxx@niaid.nih.gov; 3 Departments of Microbiology and Pathology, University of Alabama at Birmingham, Birmingham, AL 35233, USA; E-Mail: nahm@uab.edu

**Keywords:** *Streptococcus pneumoniae*, vaccine, epidemiology, serotype, pneumococcal protein

## Abstract

Seven-valent pneumococcal conjugate vaccine (PCV7) introduction and routine pediatric use has substantially reduced the burden of *Streptococcus pneumoniae* disease worldwide. However, a significant amount of disease burden, due to serotypes not contained in PCV7, still exists globally. A newly recognized serotype, 6C, was until recently, identified and reported as serotype 6A. This review summarizes the serotype epidemiology of pneumococcal disease pre- and post-introduction of PCV7, available post-marketing surveillance data following the introduction of higher valency pneumococcal vaccines (PCV10, PCV13) and future prospects for the development of new pneumococcal vaccines.

## 1. Introduction

*Streptococcus pneumoniae* is a leading cause of disease in children worldwide. Common manifestations of pneumococcal disease include meningitis, bacteremia, pneumonia and otitis media.

Development of glycoconjugate vaccines has overcome challenges to elicit protective immune responses in infants and young children. A seven-valent pneumococcal conjugate vaccine (Wyeth Pharmaceuticals Inc., Pearl River, NY, USA, Prevnar) (PCV7) is licensed in over 90 countries and is prequalified by the World Health Organization (WHO) [1]. Introduction and routine infant use of PCV7 resulted in significant reductions of invasive pneumococcal disease (IPD) among vaccinated children. A benefit of universal PCV7 use was an indirect (herd) effect, which is thought to be attributed to reduced vaccine type colonization in the nasopharynx following vaccination and, in turn, decreased transmission of these pneumococci from vaccinated to susceptible (unvaccinated) individuals. The benefits of herd immunity were in part offset by increases in nasopharyngeal colonization of serotypes not contained in PCV7 [2,3]. Replacement by non-vaccine serotypes is a concern for both the individual and the population as a whole, due to the probability of transmission and potential to cause disease.

Although routine PCV7 use has led to significant decreases in the incidence of pneumococcal disease, due to the seven vaccine types, a substantial proportion of pneumococcal disease burden, due to serotypes not included in PCV7, still exists. The selection of serotypes included in higher valency pneumococcal conjugate vaccines (PCVs) was consequently based on the need for a broader coverage of serotypes that have become more frequent causes of pneumococcal disease following PCV7 immunization, while continuing to maintain protection against disease, due to PCV7 serotypes. Currently, a 10-valent (GlaxoSmithKline Biologicals, Synflorix) (PCV10) and a 13-valent (Wyeth Pharmaceuticals Inc., Prevenar 13) (PCV13) pneumococcal conjugate vaccine is licensed and available in many countries. Synflorix contains the seven serotypes in PCV7 in addition to serotypes 1, 5 and 7F. Prevenar 13 contains the PCV10 serotypes plus serotypes 3, 6A and 19A. This review summarizes: (a) changes in the serotype epidemiology of invasive pneumococcal disease (IPD), including complicated pneumonia, prior to and following the introduction of PCV7 and higher valency PCVs; (b) available post-marketing IPD surveillance data that reflect the direct impact on the vaccinated/vaccine-eligible age group of children following the introduction of higher valency PCVs; and (c) future prospects for the development of new pneumococcal vaccines.

## 2. Changes in Serotype Epidemiology Pre- and Post-PCV7 Introduction

### 2.1. Invasive Pneumococcal Disease

The seven serotypes (14, 6B, 19F, 23F, 18C, 4, 9V) contained in PCV7 were among the most common global causes of invasive pneumococcal disease (IPD) among children <5 years of age. In many countries where PCV7 was implemented in the pediatric immunization schedule and national uptake was high, significant decreases in vaccine serotype IPD and relative increases in reported non-PCV7 serotype IPD were observed and led to a net decrease in the incidence of IPD overall [1,4,5,6]. In countries where overall PCV7 vaccine uptake was low, serotype 14 was the most frequently reported cause of IPD among the seven serotypes [1]. IPD attributable to serotypes contained in PCV10 and PCV13, respectively, globally represent approximately 75%–85% and 80%–90% of IPD among children <5 years of age [1,7]. The data presented below are mainly from studies in Europe and the Americas.

Following PCV7 introduction, serotype 19A emerged in many countries as a leading cause of non-PCV7 IPD. In 2000, PCV7 was licensed in the U.S. and recommended for all children <2 years of age and for children two years to five years of age at risk for pneumococcal disease, due to an underlying medical condition. In 1998, serotype 19A accounted for 2.5% of all invasive isolates in children <5 years of age, and in 2005, it accounted for 36% of all isolates in this age group [8]. Between 1998–1999 and 2005, the largest increase in age-specific incidence of IPD 19A occurred among children <5 years of age (6.7 cases/per 100,000 population). The proportion of serotype 19A isolates that were non-susceptible to multiple antibiotics also increased, a result partly from clonal expansion of sequence type (ST)199, during the same time period [8,9]. In 2007, 42% (*n* = 180/427) of non-PCV7 serotype IPD in children aged <5 years in the U.S. was due to serotype 19A. Serotypes 7F and 3 were responsible for the remainder of IPD caused by non-PCV7 serotypes in children aged <5 years [10]. The incidence of PCV7 serotype IPD in 2007 was <1 cases/per 100,000 population among children aged <5 years [6]. Observed increases in the incidence of serotype 19A disease are likely multifactorial, as similar trends have also been reported in countries without national immunization programs [11,12]. Other factors, such as antimicrobial use and the development of multidrug resistant serotypes, secular trends and/or the emergence of specific clones may have also contributed to changes in 19A disease rates [8,11,13].

The incidence of serotype 6A IPD in children <5 years of age was generally observed to decline after PCV7 introduction, which in part was attributed to cross-protection by 6B vaccine antigens [14]. However, serotype 6A IPD continued to be common globally in the post-PCV7 era, which may have been in part due to serotype 6C isolates that were previously identified and reported as serotype 6A [5,6,15]. Serotype 6C is a new recognized serotype within serogroup 6. Studies suggest that PCV7 does not cross-protect against serotype 6C [16,17,18]. In Europe, retrospective analyses of archived samples that were obtained from IPD surveillance before and after PCV7 introduction indicated that the prevalence serotype 6C IPD in the post-PCV7 period overall was associated with clonal expansion of the ST224-complex. The increased proportion of 6C isolates that were non-susceptible to antibiotics was concurrent with the emergence of the ST386-complex in the post-PCV7 period [19,20]. Serotype 6C PCR-screening of the archived 6A invasive disease isolates in the U.S. and Europe indicated that serotype 6C invasive disease in children is less common than in adults. However, since the magnitude of the serotype 6C absolute incidence rate increase was very small, definitive conclusions about the trends in 6C invasive disease are limited. PCV13, which contains serotype 6A and 7F vaccine antigens, has the potential to confer cross-protection against non-PCV13 serotypes 6C and 7A [21]. Opsonophagocytic antibody (OPA) titers to serotype 6C, measured from sera from infants who had participated in a PCV13 clinical trial, were ≥1:8 to serotype 6C and were measurable in 96% of PCV13 recipients. Furthermore, all of the PCV13 immune serum samples tested had detectable OPA titers ≥1:8 for both serotypes 7F and 7A [21]. Serotype 7F and 7A share serogroup-specific epitopes.

Serotype 6D has also been described within serogroup 6 [22]. Serotype 6D until recently had been identified and reported as serotype 6B [17,18,23]. To date, serotype 6D is a rare cause of pneumococcal infections in children and adults in the U.S. [20,24], but is relatively common in Asia [25].

In an international study, serotype distribution was assessed from longitudinal IPD surveillance data at sites located in Australia, New Zealand, Israel, Uruguay, North America and Canada [26]. Twenty-one surveillance databases that contained data about the rate of IPD reported for at least two years before and one year after PCV7 introduction were identified. Site-specific rate ratios (RR) were calculated using the observed IPD rate for each post-PCV7 year divided by the expected IPD rate extrapolated from the pre-PCV7 rate. For databases in which 6C isolates were differentiated from 6A, the distribution of true 6A isolates was weighted by the size of the surveillance site; 6C was included in analyses as a non-PCV7 vaccine serotype (NVT). For databases in which 6C was not reported separately from 6A isolates, the distribution of serotypes 6C and 6A was estimated from surveillance data available in the same geographic region or from global estimates reported in the pre-and post-PCV7 time periods. Among hospitalized children <5 years old, pooled analyses showed that the overall IPD rates decreased by year 1 after PCV7 introduction (RR 0.55, 95% CI 0.46–0.65) and remained relatively stable through year 7 (RR 0.49, 95% CI 0.35–0.68). Point estimates for VT IPD rates (*i.e*., PCV7 and 6A) decreased annually through year 7 (RR 0.03, 95% CI 0.01–0.10). Point estimates for rates of IPD caused by non-PCV7 serotypes contained in higher valency vaccines (*i.e.*, serotypes 1, 3, 5, 7F, 19A) were apparent by 2–3 years after PCV7 introduction (compared to the expected IPD rates for the same corresponding serotypes) and continued to increase through year 5 (RR 3.65 95% CI 2.50–5.34) [26].

In South Africa and The Gambia, PCV7 was included in the national immunization programs (NIPs) in 2008 and 2009, respectively. Serotype data from invasive disease isolates obtained during a clinical trial conducted prior to PCV7 introduction indicated that 65% of IPD cases among Gambian children aged 2–29 months resulted from serotypes contained in PCV13; serotypes 1 and 5 accounted for 18% of cases and 19A accounted for 9% of cases [27]. The most prevalent clone among serotype 1 invasive disease isolates identified from hospital surveillance or from clinical trials conducted in The Gambia between 1996 and 2005 was ST618 [28]. The incidence rate of serotype 1 IPD can vary considerably, due to secular trends and its propensity to cause epidemics. Among South African infants 1–3 months of age, 78% of IPD cases identified during a clinical trial conducted in 1998–2001 were due to serotypes contained in PCV13; serotypes 6A and 19A accounted for 14% and 9% of cases, respectively [29]. To date, longitudinal IPD surveillance data in West and South Africa following PCV7 introduction in the NIP are not available.

In Latin America, PCV7 was implemented in several NIPs in the year 2008 (Mexico, Uruguay), the year 2009 (Peru, Costa Rica) and the year 2010 (Panama, El Salvador, Ecuador). Higher valency PCVs (PCV10, PCV13) replaced PCV7 in the NIPs in Latin America mainly in the year 2011. Prior to PCV7 introduction, common serotypes in Latin America were 14, 6B, 1 and 5, as assessed in a multinational hospital-based IPD surveillance study. In Mexico, among children <5 years of age, the proportion of IPD due to serotypes contained in PCV13 decreased from 77% in the year 2007 to 65% in the year 2011; the proportion of 19A IPD reported four years after PCV7 introduction in the NIP was 3.5 fold higher than pre-PCV7 introduction (year 2007) [30]. In Uruguay, the proportion of IPD among children <5 years of age decreased from 58% (in the year 2007) to 21% (in the year 2011) for PCV7 serotypes and increased from 35% to 44% for the six additional non-PCV7 types during the same time period. The relative proportion of serotype 1 IPD among children <5 years of age in Uruguay increased from 5% in the year 2007 to 17% in the year 2011; the relative proportion of serotype 3 IPD in this age group increased from 14% during the same time period [30].

### 2.2. Pneumonia

Estimating the proportion of community-associated pneumonia (CAP) due to *S pneumoniae* is challenging. Clinical presentation of lower respiratory tract infection and chest X-ray findings are non-specific, and thus, a diagnosis of pneumococcal pneumonia based on signs and symptoms alone can be difficult to distinguish from pneumonia due to other respiratory pathogens. Culture of blood samples has low sensitivity for detecting cases of pneumococcal pneumonia and might not be routinely collected. However, the majority of CAP cases are not associated with bacteremia. In children, sputum samples are not easily obtained.

An increased occurrence of pneumococcal empyema, due to emerging serotypes, and the continued prominence of serotype 1 as a cause of severe pneumonia post-PCV7 introduction support the added potential benefit of higher valency pneumococcal vaccines to prevent a greater proportion of pneumonia cases, including pneumonia associated with complications. Pneumonia complicated by the development of empyema (alone or with parapneumonic effusion) increased among hospitalized children with pneumonia since the late 1990s [31,32,33]. Before the availability of PCV7, data from tertiary hospitals in the U.S. and Europe showed that empyema due to *S. pneumoniae* among hospitalized children with pneumonia was predominately due to serotype 1, with serotype 14 also identified as a common cause [31,32,33]. Following the introduction of PCV7 in infants and young children, the rates of pediatric IPD due to empyema increased, due to relative increases in cases caused by non-PCV7 serotypes [32,34]. In a study conducted at a tertiary hospital in the U.S., the incidence of empyema was 8.5/100,000 prior to the PCV7 introduction and increased to 12.5/100,000 after PCV7 introduction. At the time the study was conducted, 88% percent of children in Utah had received three doses of PCV7 by 35 months of age. The proportion of empyema caused by non-PCV7 serotypes increased from 62% to 98% during the study period, with serotype 1 reported as a prominent cause of empyema before and after PCV7 introduction (50% and 33%, respectively) and serotypes 3 (27%) and 19A (26%) identified as emerging causes of severe pneumonia post-PCV7 introduction. Molecular analyses indicated that the sequence type representing each of three serotypes (1, 3, 19A) was present before PCV7 introduction, suggesting that the increase in the incidence of empyema was a result of serotype replacement by non-PCV7 serotypes [32].

Similar to the U.S., the prominence of serotype 1 and its association with pneumococcal pneumonia complicated by empyema was observed prior to PCV7 introduction in Europe. The emergence of other non-PCV7 types accounting for complicated pneumonia reported in Europe was noted during the time period after PCV7 introduction, as well [35,36]. In a study conducted in Italy, empyema or other pneumonia complications (e.g., parapneumonic effusion) was reported in 162 of 753 (21.5%) children hospitalized with pneumonia. At the time of the study, 41% of the participants had been vaccinated with PCV7 prior to enrollment. Of the 80 identified pneumococcal bacteremic pneumonia cases, >67% of cases were due to non-PCV7 types, with 32.5%, 15% and 12.5% of cases attributed to serotype 1, 19A and 3, respectively. Of children with complicated pneumococcal pneumonia, 50% were associated with serotype 1. All cases of serotype 1 pneumococcal pneumonia occurred in children older than two years of age, while pneumonia due to 19A was common in younger children (median age: 3.1 years (range: 10 months to 3.7 years)). All of the pneumonia cases due to PCV7 serotypes occurred in children who were not vaccinated with PCV7. In a study conducted at a pediatric tertiary hospital in Spain, the most common cause of pneumococcal parapneumonic empyema was serotype 1 (42%) followed by serotypes 7F, 3, 19A and 5. Forty-six percent of children had received at least one dose of PCV7 [36].

## 3. Implementation of Higher Valency Pneumococcal Conjugate Vaccines (PCV10, PCV13)

### 3.1. Serotype Coverage

In Europe and North America, the serotypes included in PCV10 and PCV13 are estimated to cover approximately 80%–85% and 85%–90% of IPD, respectively, in children <5 years of age; serotypes 19A, 7F, 1 and 6A are prominent causes of IPD in regions that previously included PCV7 in the NIP [1,7,37]. In Africa, serotypes 1, 5 and 6A are common causes of IPD, and both PCV10 and PCV13 are estimated to cover >70% of the serotypes [7].

In Latin America, PCV7 was not widely used, and IPD caused by PCV7 serotypes currently represent approximately 55%–60% of the disease burden [7]. Use of PCV10 or PCV13 would potentially increase coverage to approximately 75% and 80%, respectively [7]. In Latin American countries that have included PCV7 in their NIP, IPD due to serotypes 1, 5 and 6A are becoming more prominent [1].

### 3.2. Epidemiology of Vaccine Serotype IPD Following the Introduction of 10-Valent and 13-Valent PCVs

PCV10 and PCV13 were first approved in 2008 and 2009, respectively. Currently, both PCV10 and PCV13 are approved in >100 countries and prequalified by WHO [7,38]. The most commonly administered regimens include two or three doses in infancy followed by one dose in the second year of life (2 + 1, 3 + 1) or three infant doses with no toddler dose (3 + 0) [1,39].

In Germany, PCV10 and PCV13 were introduced in the NIP in April, 2009, and December, 2009, respectively, which was approximately three years after PCV7 introduction [1,38]. PCV10 and PCV13 are administered at two, three, four and 11–14 months of age. At the time that PCV13 was introduced in the NIP, approximately 50% of children had been immunized with PCV10. By 2011, approximately 85 of children were receiving PCV13. Prior to the introduction of PCV10, 37% and 68% of IPD in children <2 years of age were caused by serotypes contained in PCV10 and PCV13, respectively. The most common serotypes pre-PCV10 introduction were 7F, 19A and 1. In 2011, approximately two years after PCV13 introduction, six cases of IPD due to non-PCV7 serotypes (1, 3, 5, 6A, 7F, 19A) were reported in children <2 years of age compared to 28 cases reported in 2009. Reports of serotype 19A IPD decreased from 15 cases in 2009 to one case in 2011 [40].

In the United Kingdom (UK), PCV13 replaced PCV7 in the national immunization program (NIP) in April, 2010, which was approximately four years after routine infant immunization with PCV7 began (September 2006). PCV13 was administered at two, four and 12 months of age. Prior to PCV13 introduction in the NIP (September 2009, to April 2010), the proportion of IPD cases due to the six non-PCV7 types was 69% (*n* = 191/277) compared to 27% (*n* = 82/303) of non-PCV7 types present in the first year after PCV7 introduction. The most prevalent serotype pre-PCV13 introduction was 19A followed by serotype 7F and 1 [41]. Approximately three years after the introduction of PCV13, the cumulative weekly cases of IPD caused by the six additional serotypes in PCV13 in children <2 years of age decreased to approximately the same as the number of cases prior to the introduction of PCV7 [42]. Decreases in the number of cumulative cases were mainly due to 19A and 7F [43].

In the U.S., PCV13 replaced PCV7 in the routine infant schedule in approximately March, 2010. Infants received PCV13 at two, four, six and 12–15 months of age. Furthermore, a supplemental PCV13 dose was administered to children who were fully vaccinated with PCV7. Preliminary IPD surveillance data (April, 2010, to March, 2011) indicated trends towards reduced rates of 19A and 7F IPD in children <2 years old, compared to matched calendar quartiles during a time period (2006–2008) prior to PCV13 introduction. During the first year after PCV13 introduction, there was no change in the rates of serotype 3 IPD and too few cases of serotype 1, 5 and 6A to evaluate any potential effects [44].

In Latin America, several countries included PCV10 or PCV13 as the first PCV in their NIPs in the year 2010 (Brazil, Columbia), the year 2011 (Chile, Nicaragua, Honduras) or the year 2012 (Argentina, Paraguay). In Chile, the proportion of PCV10 serotype IPD among children <5 years of age declined from 74% (*n* = 209/282) in the year 2010 to 52% (*n* = 88/168) in the year 2012. In five countries, PCV7 was introduced in the NIP during 2009–2010 and then replaced by PCV10 (Peru, Ecuador) or PCV13 (Costa Rica, Panama, El Salvador) in the year 2011. In Mexico, PCV7 was introduced in the NIP in 2008 and replaced by both PCV10 and PCV13 (year 2010 and 2011, respectively). The number of IPD cases due to the six non-PCV7 serotypes that occurred among Mexican children <5 years of age declined from 40% (*n* = 49/124) in 2008 to 13% (*n* = 14/105) in 2012 [30].

## 4. Non-PCV13 Serotypes: Epidemiology Pre-and Post-Introduction of Higher Valency PCVs

### 4.1. Interim Time Period after PCV7 Introduction and before the Introduction of Higher Valency PCVs

During the interim period following PCV7 introduction, but prior to PCV13 introduction, decreases in PCV7-serotype IPD in countries with established PCV7 immunization programs were also associated with relative increases in IPD, due to serotypes not covered by currently available multivalent PCVs (PCV10, PCV13), such as 22F, 33F, 15B/C and 11A [24,45,46,47].

In the U.S., PCV7 was recommended for routine infant immunization in 2000. In 2006–2007, serotypes 22F and 33F and 15B/C were the most common causes of non-PCV13 serotype IPD among children <5 years of age and cumulatively accounted for 14% of IPD in this age group overall. In contrast, prior to PCV7 introduction (1998–1999), serotypes 22F, 33F and 15B/C altogether accounted for 2.5% of IPD among children <5 years of age [6]. Four non-PCV13 serotypes comprised a cumulative total of 32% of the penicillin nonsusceptible (PNS) isolates in 2007: 15A (11%), 23A (8%), 35B (8%) and 6C (5%) [45].

In Europe, serotype 22F was one of the most frequently reported non-PCV13 serotypes in 2010 among children <5 years of age [24]. In Norway, PCV13 replaced PCV7 as a routine childhood vaccine starting in 2010. Prior to PCV13 introduction (2007 to 2009), serotypes 22F, 15B/C and 38 were among the increasing causes of non-PCV13 serotype IPD. During the same time period, there was rapid clonal expansion of ST433, ST199, and ST393, respectively. The emergence of serotypes 15 B/C was associated with the expansion of other clones; however, the most common sequence type continues to be ST199 [48]. In the UK, PCV7 was introduced in 2006. During the time period between 2000–2006 and 2008–2010, rates of IPD were assessed from cases identified through national surveillance. The analyses of cases took into account potential biases due to missing data (serotype and age of patient) and changes in case ascertainment, such as case identification via molecular methods and routine screening for serotype 6C isolates, during the surveillance period. In 2008–2010, the average numbers of non-PCV13 serotype IPD cases reported among children <5 years of age were highest for serotypes 22F, 15B/C and 33F (*n* = 34, 22 and 15 cases, respectively). During the time period from 2000–2006 to 2008–2010, the incidence rate (adjusted for potential biases) of IPD for non-PCV13 serotypes 22F and 15C increased by approximately three-fold among children <5 years of age [15].

In Australia, PCV7 was recommended in 2005 for non-Aboriginal children as a three-dose infant schedule (ages two, four and six months) without a toddler dose. Vaccine coverage was 88% among non-Aboriginal children in 2005. In a study conducted in Western Australia, the overall incidence of IPD in children <5 years of age decreased in 2005–2007 compared to 2002–2004, and an increase in the frequencies of non-PCV13 serotype IPD (6% to 17%) was reported during the same time period. The most common non-PCV13 serotypes in 2007 were 15B/C and 11A [49]. In contrast, in a study conducted in Southern Australia, no substantive increases in non-PCV13 serotypes were reported in children <5 years of age during the time period from 2002–2004 to 2007–2009 [50].

In Canada, a national surveillance study was conducted during 2007–2009 to assess the baseline epidemiology prior to PCV13 introduction. Of the 800 invasive disease isolates obtained during the surveillance period, serotypes 22F (6.0%) and 11A (4.4%) were the most common non-PCV13 serotypes overall (all age groups). The proportions of serotype-specific IPD varied by age group (<2 years, three to 16 years, 17 to 49 years, ≥50 years). Among children <2 years of age, the most common non-PCV13 serotypes causing IPD included 15B (10.0%), 23A (6.7%), 22F (5.0%) and 35B (5.0%) [47].

### 4.2. After PCV13 Introduction

In a case-based study conducted at eight pediatric hospital centers in the U.S., invasive disease isolates were prospectively identified during a surveillance period beginning from January 1, 2007, through December 31, 2011. In 2010–2011 (post-PCV13 introduction), non-PCV13 serotypes 33F (*n* = 16) and 22F (*n* = 12) followed by serotypes 12, 15B, 15C and 23A (*n* = 7 for each of the serotypes) were the most common causes of invasive pneumococcal infection. In total, the six serotypes described above accounted for 20% of cases per year in 2010 and in 2011, compared to 10% of cases per year in 2007–2008 (post-PCV7 introduction, but prior to PCV13 introduction). Serogroup 11 accounted for six IPD cases in 2011, compared to zero to four serotype 11 IPD cases/per year in 2007–2010 (pre-PCV13 introduction) [51].

## 5. Future Prospects for New Pneumococcal Vaccines

In light of the present epidemiology of pneumococcal disease, PCV10 or PCV13 immunization in infants has the potential for significantly reducing the global pneumococcal disease burden that exists today. However, invasive disease serotypes not covered by currently available PCVs are already evident [15,45,47,49] and might become prominent causes of reported disease as circulating vaccine invasive serotypes decrease in countries using PCV10 or PCV13. In turn, a 15-valent PCV, which includes serotypes 22F and 33F, is being developed to offset some of the projected replacement serotypes that are anticipated to accompany routine PCV10 or PCV13 use [52]. In the long run, as the geographic distribution of predominant serotypes changes, effective vaccine coverage provided by PCVs may not be optimal worldwide. Furthermore, manufacturing complexity and the high cost of PCVs limit the ability to sustain production in developing countries. Alternative strategies for the development of serotype-independent pneumococcal vaccines that include common proteins are underway. While many potential choices for vaccine antigens are in the preclinical stages of development, there are a growing number of investigational pneumococcal protein-based vaccines that have recently been or are currently being evaluated in clinical trials [53,54,55,56,57]. General categories of protein-based vaccines in development include serotype-independent subunit vaccines comprised of purified proteins, proteins antigens that are expressed by recombinant bacteria, combination vaccines that include pneumococcal protein antigens in addition to conjugate components and a whole-cell vaccine.

### 5.1. Protein-Based, Serotype-Independent Subunit Vaccines

Protein-based, serotype-independent subunit vaccines could circumvent the issue of serotype replacement by directly targeting proteins that are highly conserved among a diversity of pneumococcal serotypes. Many of the vaccine candidates that have been studied are proteins that are involved in pathogenesis and can be present on the surface of intact pneumococci or a component that contributes to cell lysis. A noncapsular protein-based vaccine that contains several pneumococcal proteins in a single formulation could potentially provide broader protection, for example, if antibodies to each of the proteins antigens confer protective immunity through one or more mechanism or if the proteins are expressed at different stages of pathogenesis.

Investigational multicomponent protein-based subunit vaccines that contain combinations of pneumococcal surface protein A (PspA), pneumococcal choline-binding protein A (PcpA) and PhtD (polyhistidine triad protein D) have recently been or are currently being evaluated in Phase 1 clinical trials [53,56]. A phase 1 clinical trial designed to evaluate the safety and immunogenicity of a DNA vaccine containing a PspA gene expressed in an attenuated *Salmonella typhi* vector has been completed, as well [58]. PspA is among the earliest studied virulence factors. Although PspA is structurally variable, it is expressed on the surface of many clinically relevant pneumococcal strains. Cross-protection against strains expressing heterologous PspAs has been shown in pre-clinical studies [59]. PcpA is thought to have a role in adherence to host cells, particularly respiratory epithelium, which would be a favorable characteristic in light of the increased attention to pneumococcal pneumonia prevention. Polyhistidine triad protein D (PhtD) is expressed on the surface of many pneumococcal serotypes and has an amino acid sequence that is highly conserved. Antibodies elicited to individual Pht proteins (PhtA, PhtB, PhtD and PhtE) are cross-reactive with antibodies to other proteins within the Pht family [60]. Possible functions of Pht proteins in pathogenesis include adhesion to host cells and downregulation of the complement pathway by binding to factor H. In animal models, immunization with PhtD in combination with other protein antigens has been shown to reduce nasopharyngeal colonization and protect against pneumococcal infection, including pneumonia and invasive disease [61]. Another conserved virulence factor, pneumolysin, is a known intracellular toxin that has also been shown in animal sepsis and pneumonia models to have an enhanced protective effect when given in combination with other proteins [62]. Derivatives of pneumolysin toxoid have been or are currently being evaluated in clinical trials (denoted as dPly or PlyD1 hereafter) [55,56].

### 5.2. Combination (Protein Vaccine Antigens Plus PS-Conjugates) Vaccine

A combined vaccine that includes pneumococcal proteins as vaccine antigens in addition to polysaccharide-conjugated components is another possible approach to broaden the scope of protection. In an animal model, anti-PhtD antibodies in conjunction with anticapsular antibodies had an additive protective effect against sepsis following intranasal challenge, compared to the effect elicited by anti-capsular antibodies alone [63]. An investigational vaccine containing PhtD, dPly antigens and pneumococcal polysaccharide-conjugated components is in development, and a phase 2 trial was conducted to evaluate the safety and immunogenicity of this vaccine in children 12–23 months of age [64]. An additional role of pneumococcal protein(s) might be as a carrier protein(s) for conjugated vaccine components.

### 5.3. Whole-Cell Vaccine

A pneumococcal whole-cell vaccine (WCV) comprised of killed *S. pneumoniae* organisms enable the simultaneous presentation of multiple surface protein antigens. A vaccine that could provide direct protection against pneumococcal disease and possible indirect protection by reducing nasopharyngeal colonization is an approach that takes into consideration the capability of a WCV to elicit both humoral and cellular-mediated immune responses. In animal models, a WCV derived from killed unencapsulated bacterium was shown to induce antibodies that could passively protect against lethal challenge and to stimulate IL-17A-mediated responses, leading to reduced density of pneumococcal colonization in the nasopharynx and middle ear [65,66]. A phase 1 clinical trial with the objective of evaluating the safety of an aluminum-adjuvanted WCV was completed in 2013 [57].

## 6. Conclusions

PCV7 has been highly effective in reducing global pneumococcal disease burden. In countries where PCV7 was implemented and uptake has been high, significant decreases in vaccine serotype IPD and relative increases in reported non-PCV7 serotype IPD have been observed. The net decreases in the overall incidence of IPD strongly support the benefits of PCV7 vaccination.

The magnitude of increased rates of pneumococcal non-vaccine serotype invasive disease reported after the implementation of PCV7 varied among geographic regions and by the extent of vaccine uptake. While shifts in epidemiology temporally followed PCV7 introduction, the extent to which the changes were due to vaccination is unclear. Available baseline pre-PCV7 IPD surveillance data are limited, which makes it difficult to discern if the changes in serotype distribution primarily represented true replacement disease or resulted from non-vaccine factors, such as secular trends or antibiotic selection pressure. Changes in surveillance methods or vaccine uptake could also lead to artificial fluctuations in overall IPD incidence. Ultimately, it remains uncertain to what extent an increase in the prevalence of non-PCV7 serotypes would translate to complete replacement of pneumococcal disease by these serotypes, compared to the overall rates of clinical disease caused by PCV7 vaccine types.

A significant proportion of pneumococcal IPD that globally exists today can be prevented by the extended serotype coverage provided by PCV10 and PCV13. Importantly, the introduction of PCV10 and PCV13 can potentially have a broader impact on pneumococcal CAP, as serotypes contained in both vaccines represent serotypes that are prominent causes of bacterial pneumonia in children and adults. Preliminary surveillance data post-PCV10 and PCV13 introduction indicate trends towards a decreased incidence of IPD due to 19A and 7F among vaccinated children. Ongoing surveillance is essential to assess the effectiveness of PCV10 and PCV13 to prevent PCV7 and non-PCV7 serotype IPD. Long-term surveillance is likewise necessary to monitor for emerging and increasingly prevalent pneumococcal invasive serotypes, including non-PCV13 serotypes, such as 15B/C, 22F, 33F and 23A.

Protein-based pneumococcal vaccines potentially can overcome some of the limitations of polysaccharide conjugate vaccines. In concept, broader vaccine protection could be achieved via inducing antibodies to protein antigens that are conserved among a diverse number of serotypes, as well as proteins that significantly contribute to pathogenesis. Pre-clinical data with candidate vaccines based on subunit protein antigens, combinations of conjugate and pneumococcal protein antigens or whole-cell pneumococci indicate that antibodies elicited by candidate protein antigens can prevent pneumococcal infections in animal models of invasive and mucosal disease. An increasing number of candidate protein-based pneumococcal vaccines have recently been or are currently being evaluated in phase 1 and phase 2 clinical trials. Additional studies are needed to further evaluate how vaccine-induced changes to pneumococcal serotype distribution in the nasopharynx affect disease transmission potential and long-term impact on the incidence of pneumococcal disease.
